# Editorial: Temperature-dependent mechanisms of neuron functioning: Emerging concepts

**DOI:** 10.3389/fncel.2022.1009071

**Published:** 2022-08-18

**Authors:** Sergiy M. Korogod, Merab Tsagareli, Patrick Delmas, Alexander V. Zholos

**Affiliations:** ^1^Department of Molecular Biophysics, O. O. Bogomoletz Institute of Physiology, National Academy of Sciences of Ukraine, Kyiv, Ukraine; ^2^Laboratory of Pain and Analgesia, Beritashvili Center for Experimental Biomedicine, Tbilisi, Georgia; ^3^SomatoSens, Laboratory for Cognitive Neuroscience, Centre National de la Recherche Scientifique, UMR7291, Aix-Marseille University, Marseilles, France; ^4^Department of Biophysics and Medical Informatics, ESC “Institute of Biology and Medicine”, Taras Shevchenko National University of Kyiv, Kyiv, Ukraine

**Keywords:** ion channels, temperature, neurons, excitability, spiking patterns, thermosensing mechanisms

Life of organisms crucially depends on ability to detect external and internal temperatures and adequately react to their variations. This ability is provided by multiple mechanisms operating in neurons and non-neuronal cells at different structural levels, from molecular and sub-cellular to cellular and systemic. Studies of such mechanisms in various research domains each year bring new findings, which require an integrated comprehensive assessment. A pledge of progress in the field is establishing closer mutually enriching inter-domain links. This Research Topic is designed as a step forward on this way. It is aimed at assembling contributions of researchers from different domains of expertise and building together a conceptual framework for deeper insight into mechanisms underlying temperature-dependence of functioning of neurons and other cell types in health and disease. It contains 6 contributions including 3 original research articles, 2 reviews, and 1 mini review.

Brain temperature has been reported to influence sensory processing, working memory, motor and other functions. This factor is important in clinical context, e.g., local brain cooling effectively suppressed foci of drug-resistant pathological activity in epileptic patients. Gotoh et al. found that cortical evoked potentials (CEPs) in anesthetized rats decreased in amplitude to nearly zero when the local brain temperature decreased below 17°C and, in contrast, increased with decreasing temperature from near-physiological level of 36°C to >17°C. The negative correlation of CEPs and local cortical temperatures remaining above 17°C was eliminated by the administration of a GABAA receptor antagonist. It is suggested that the observed negative correlation is caused by an alteration of the balance of contributions of excitatory and inhibitory inputs to the CEPs, possibly due to higher temperature sensitivity of inhibitory inputs. This finding highlights the necessity for further exploring the temperature dependency of excitatory and inhibitory mechanisms underlying the mediation of neural information processing by temperature.

Thermal challenges can severely affect the behavior and survival of animals by disrupting neuronal activity and coordination. The survival critically depends on possessing compensatory mechanisms that protect neurons from detrimental temperatures. In crustacean stomatogastric ganglion (STG), such compensation is important for coordination of circuits generating the slow gastric mill and fast pyloric rhythms that is maintained across a broad temperature range. Städele and Stein explored the underlying mechanisms in crab *Cancer borealis* and found that temperature robustness and coupling between the mentioned two oscillator circuits are enabled by neuromodulation originating from modulatory commissural neurons. In particular, it was a peptidergic modulatory projection neuron MCN1, which sits in a commissural ganglion (CoG) and innervates the gastric mill circuit. The gastric mill rhythm disappeared when STG was individually heated to a certain “crash” temperature, and it was restored when upstream CoGs were also heated to the crash temperature. Heating increased the activity of MCN1 and its neuropeptide transmitter stabilized and maintained the rhythm over a broad temperature range. The authors concluded that extrinsic neuromodulation is essential for the STG oscillatory circuits and maintaining their function in temperature compromised conditions.

In the article by Buijs and McNaughton, the role of cold-sensitive TRP channels (TRPM8, TRPC5, and TRPA1) and cold-sensitive ion channels in sensory neurons are reviewed including two-pore domain potassium channel (K2P), glutamate receptor (GluK2), and cyclic nucleotide-gated ion channel (CNGA3). They explore periphery cold sensation in the sensory nerve endings that can produce moderate innocuous cold on the one hand, and noxious cold sensation on the other hand. The latter triggers withdrawal reflexes allowing animals to avoid damage.

In their review Rueda-Ruzafa et al. discuss the importance of the potassium channels of the TREK subfamily, belonging to the recently discovered family of two-pore domain potassium channels (K2P) in the transduction of thermal sensitivity in different cell types. When the temperature is reduced (from 30 to 10 °C), TREK channel activity would fall to zero and the activity of cold sensitive TRPs would strongly increase. At this point, both channels tend to depolarize the cold thermoreceptor which in turn should increase firing and send the information to the next step. It seems clear that both TREK1 and TRAAK are important to regulate the excitability of DRG thermosensitive neurons as removal of both is necessary to observe a clear effect in the process of cold sensing.

Although it is such an important feature, our knowledge regarding the fundamental principles of thermosensation remains incomplete. The ion channel TRPV1, a member of the vanilloid TRP family of non-selective cation channels, is involved in a wide range of processes including nociception and thermosensation. TRPV1 is highly expressed in nociceptors within dorsal root and trigeminal sensory ganglia. TRPV1 was found to be activated by elevated temperatures (≈43°C) that evokes pain in vivo. Additional studies demonstrated that TRPV1 can alternatively be activated by a range of chemical stimuli (e.g., capsaicin, proton) and further sensitized by pro-inflammatory substances. However, there is accumulating evidence that TRPV1 is expressed in a multitude of non-neuronal sites and have functional roles away from sensory nerve activity. The contribution by Shuba shed light onto very different functions of TRPV1 and discusses the expression, functionality, and roles of these non-neuronal TRPV1 channels in addition to a direct role in pain and neurogenic inflammation.

Many TRP subtypes are voltage-gated ion channels directly or indirectly regulated by intracellular calcium. These properties allow their efficient cross-talk with other channel types *via* changes in membrane potential and/or intracellular calcium concentration. However, the functional outcomes of such interactions are far from clear. Maksymchuk et al. developed a quantitative mathematical/biophysical model that accounted for parallel activities of six different types of channels, including thermo-TRPs, voltage-gated Na^+^, K^+^, and Ca^2+^, and small- and large-conductance Ca^2+^-activated K^+^ channels in *Drosophila* larva cold-sensing Class III somatosensory neurons. This comprehensive model allowed the authors to explain exactly how neuronal spiking rate changed depending on the rate of cooling, the final level of cold temperature and direction of temperature change (i.e., decrease or increase of temperature). The authors conclude that the same primary cold nociceptors can encode all three modalities of cold sensing.

## Author contributions

All authors listed have made a substantial, direct, and intellectual contribution to the work and approved it for publication.

## Funding

This work was supported by the National Academy of Sciences of Ukraine (Project No. 0118U007347 to SK), the CNRS, Aix-Marseille Université, and the European Commission ERA-NET scheme (to PD), and the Ministry of Education and Science of Ukraine (Grant No. 0122U001535 to AZ).

## Conflict of interest

The authors declare that the research was conducted in the absence of any commercial or financial relationships that could be construed as a potential conflict of interest.

## Publisher's note

All claims expressed in this article are solely those of the authors and do not necessarily represent those of their affiliated organizations, or those of the publisher, the editors and the reviewers. Any product that may be evaluated in this article, or claim that may be made by its manufacturer, is not guaranteed or endorsed by the publisher.

